# Radiolabeling Human Peripheral Blood Stem Cells for Positron Emission Tomography (PET) Imaging in Young Rhesus Monkeys

**DOI:** 10.1371/journal.pone.0077148

**Published:** 2013-10-03

**Authors:** Alice F. Tarantal, C. Chang I. Lee, David L. Kukis, Simon R. Cherry

**Affiliations:** 1 Department of Pediatrics, University of California Davis, Davis, California, United States of America; 2 Department of Cell Biology and Human Anatomy, University of California Davis, Davis, California, United States of America; 3 California National Primate Research Center, University of California Davis, Davis, California, United States of America; 4 Department of Radiology, University of California Davis, Davis, California, United States of America; 5 Center for Molecular and Genomic Imaging, University of California Davis, Davis, California, United States of America; 6 Department of Biomedical Engineering, University of California Davis, Davis, California, United States of America; Banner Alzheimer's Institute, United States of America

## Abstract

These studies focused on a new radiolabeling technique with copper (^64^Cu) and zirconium (^89^Zr) for positron emission tomography (PET) imaging using a CD45 antibody. Synthesis of ^64^Cu-CD45 and ^89^Zr-CD45 immunoconjugates was performed and the evaluation of the potential toxicity of radiolabeling human peripheral blood stem cells (hPBSC) was assessed *in vitro* (viability, population doubling times, colony forming units). hPBSC viability was maintained as the dose of ^64^Cu-TETA-CD45 increased from 0 (92%) to 160 µCi/mL (76%, *p*>0.05). Radiolabeling efficiency was not significantly increased with concentrations of ^64^Cu-TETA-CD45 >20 µCi/mL (*p*>0.50). Toxicity affecting both growth and colony formation was observed with hPBSC radiolabeled with ≥40 µCi/mL (*p*<0.05). For ^89^Zr, there were no significant differences in viability (*p*>0.05), and a trend towards increased radiolabeling efficiency was noted as the dose of ^89^Zr-Df-CD45 increased, with a greater level of radiolabeling with 160 µCi/mL compared to 0–40 µCi/mL (*p*<0.05). A greater than 2,000 fold-increase in the level of ^89^Zr-Df-CD45 labeling efficiency was observed when compared to ^64^Cu-TETA-CD45. Similar to ^64^Cu-TETA-CD45, toxicity was noted when hPBSC were radiolabeled with ≥40 µCi/mL (*p*<0.05) (growth, colony formation). Taken together, 20 µCi/mL resulted in the highest level of radiolabeling efficiency without altering cell function. Young rhesus monkeys that had been transplanted prenatally with 25×10^6^ hPBSC expressing firefly luciferase were assessed with bioluminescence imaging (BLI), then 0.3 mCi of ^89^Zr-Df-CD45, which showed the best radiolabeling efficiency, was injected intravenously for PET imaging. Results suggest that ^89^Zr-Df-CD45 was able to identify engrafted hPBSC in the same locations identified by BLI, although the background was high.

## Introduction

In vivo imaging techniques with sufficient sensitivity to detect small quantities of cells are needed to determine the safety and efficiency of new stem/progenitor cell therapies for a spectrum of human diseases. A crucial gap for effective in vivo imaging technologies in stem cell research is the need to improve detection sensitivity, and to ensure that images can accurately identify transplanted cells at a given anatomical location. Nuclear medicine techniques, particularly positron emission tomography (PET), have much higher sensitivity than magnetic resonance imaging (MRI), and unlike bioluminescence imaging (BLI), which has similar sensitivity, can provide three-dimensional quantitative images. Outcomes with PET can also be translated from animal models to humans since many of the radiotracers are currently used in a human clinical setting [1]–[4].

We previously reported techniques for radiolabeling stem and progenitor cells with ^64^Cu- pyruvaldehyde-bis(N4-methylthiosemicarbazone) (PTSM) for PET imaging [5]. Studies showed that a minimum of 2.5×10^4^ CD34+ hematopoietic cells (1.1 pCi/cell) and 6.25×10^3^ mesenchymal stromal cells (4.4 pCi/cell) could be detected, and that each cell type had a different level of sensitivity to the radiolabeling technique. ^64^Cu has a half-life of 12.7 hours allowing cells to be tracked for approximately 2–3 days, and we demonstrated a radiolabeling dose that avoided cell toxicity. We also adapted this method to radiolabel and track transplanted renal precursors differentiated from human embryonic stem cells in fetal rhesus monkeys in vivo [6]. These studies showed effective radiolabeling techniques for renal precursors without toxicity, and correlative imaging outcomes with PET, BLI, and at the tissue level.

The overall goal of the current studies was to explore a new radiolabeling method for PET imaging specifically related to human peripheral blood stem cells (hPBSC) as a prototype cell population. We also utilized an established rhesus monkey model of prenatal hematopoietic stem cell transplantation [7] for preliminary in vivo studies. Direct radiolabeling of cells provides the best contrast-to-noise environment for cell detection because there is essentially no background when the majority of detected signal comes from the cells. Since all of the reagents and techniques proposed are specific for human cells, the methodologies can be directly translated to humans. In these studies we investigated both ^64^Cu and ^89^Zr using a radioimmunoconjugate technique that targets the cell surface instead of an internalized agent such as PTSM, which we have previously used. ^89^Zr has a half-life of 3.3 days providing an opportunity to monitor cells for longer time periods when compared to ^64^Cu, and also provides a more favorable decay profile (22.7% positron emission, 77.3% electron capture). Since tracking of radiolabeled cells is limited by half-life we also considered a new way to begin to assess engraftment by targeting a cell-surface marker in vivo, specifically CD45 (common leukocyte antigen). Our primary objectives were to first effectively synthesize the radioimmunoconjugates using CD45, to test the safety and efficiency of radiolabeling hPBSC and identify an effective radiolabeling paradigm, then to assess the ability to identify transplanted hPBSC in the monkey model. We utilized PET imaging in vivo in these studies in two ways: post-injection of radioimmunoconjugated hPBSC to track the injected cells, and after direct injection of the radioimmunoconjugate with the goal of homing to engrafted hPBSC. The results of these studies have shown new methods for synthesizing ^64^Cu-CD45 and ^89^Zr-CD45 immunoconjugates and optimum radiolabeling methods for PET imaging.

## Materials and Methods

### Animals and Ethics Statement

All animal protocols were approved prior to implementation by the Institutional Animal Care and Use Committee (IACUC) at the University of California, Davis, and all procedures conformed to the requirements of the Animal Welfare Act. Activities related to animal care including housing, feeding, and environmental enrichment were performed in accordance with IACUC-approved standard operating procedures (SOPs) at the California National Primate Research Center (http://www.cnprc.ucdavis.edu). No animals were euthanized for the imaging studies described. Normally cycling, adult female rhesus monkeys (*Macaca mulatta*) (N = 6) with a history of prior pregnancy were bred and identified as pregnant, using established methods [8]. Activities related to animal care (diet, housing) were performed according to Primate Center IACUC-approved SOPs. Fetuses were transplanted prenatally with human male donor hPBSC (25×10^6^ cells/fetus) using an intraperitoneal approach as previously described, monitored sonographically during gestation, and newborns delivered by cesarean-section at term then raised in the nursery according to established protocols for postnatal studies [7], [8]. PET imaging studies were conducted when animals were approximately 3 months postnatal age.

### hPBSC

CD34+ hPBSC were obtained from a commercial source (AllCells, Berkeley, CA) and cryopreserved using a controlled rate cryopreservation protocol in aliquots as previously reported [7]. All hPBSC used in these studies were from the same male donor.

### Transduction

Aliquots of cells used for transplantation were thawed and plated in non-tissue culture-treated 25 cm^2^ flasks coated with 4 µg/cm^2^ of the RetroNectin (Takara Bio Inc., Kyoto, Japan). hPBSC were incubated overnight in X-Vivo 15 serum-free medium (Lonza, Hopkinton, MA) supplemented with 2 mM L-glutamine, 100 units/mL penicillin, and 100 µg/mL streptomycin, and containing 100 ng/mL of thrombopoietin (TPO), Flt3-Ligand (Flt3-L), and stem cell factor (SCF) (R&D Systems, Minneapolis, MN). Cells were resuspended in fresh medium containing cytokines and transduced with an HIV-1-derived lentiviral vector expressing firefly luciferase under the control of the MND promoter at a final vector concentration of 8×10^7^ TU/mL, with protamine sulfate at 4 µg/mL (American Pharmaceutical Partners, Inc., Schaumburg, IL) using established methods [6]. After 2 h, the medium volume was doubled and cells were incubated at 37°C with 5% CO_2_ for 6–8 h. A second aliquot of the lentiviral vector was added to the cells and incubated overnight. Cells were washed with fresh medium and incubated overnight, then washed and cultured in fresh medium for 2 days. Prior to transplantation, cells were washed in sterile phosphate buffered saline (PBS) three times, and cell counts and viability were determined using trypan blue exclusion.

### Radiochemistry

A mouse anti-human CD45 antibody (BD Biosciences, San Jose, CA) was used for these studies. Bromacetoamido benzyl TETA (BAT) was kindly provided by Claude F. Meares, University of California, Davis. ^64^Cu was obtained from Washington University and ^89^Zr from IBA (Belgium). Copper (II) chloride (Fisher Scientific, Pittsburgh, PA) and zirconium (IV) chloride (Sigman-Aldrich, St. Louis, MO) were used for carrier added experiments. Sephadex G50 (Sigma) was used for molecular sieving filtration and 0.22 µm SLGV syringe end filters (Millipore, Billerica, MA) were used for final filtration of radiolabeled antibody. All solvents and buffer reagents were reagent grade or better, purchased from commercial sources, and used without additional purification. Aqueous buffers were prepared with 18 mΩ purified water (Millipore). Isocratic molecular sieving HPLC (Waters, Milford, MA) was performed with a 300×7.8 mm 5 μ Biosep SEC-2000 column (Phenomenex, Torrance, CA), eluted in 0.1 M sodium phosphate (aq), pH 6.8, at 1.5 mL per minute, with UV (280 nm) and radioanalytic (Bioscan, Inc., Washington, DC) detection. In this system, radiolabeled antibody eluted at 6.4 min, and ^64^Cu- and ^89^Zr-EDTA eluted at 9.8 min.

Mouse anti-human CD45 antibody was conjugated to the copper-chelating agent TETA by combining antibody, 2-iminothiolane (2IT) (Sigma), and BAT at concentrations of 0.07 mM, 1.0 mM, and 2.0 mM, respectively, in 0.1 M ammonium phosphate, pH 9 (0.25 mL) for 1 h at 37°C. TETA-CD45 was purified and transferred to 0.1 M ammonium acetate, pH 5, by molecular sieving filtration. Three TETA molecules were conjugated per antibody, as determined by copper-binding assay with carrier-added ^64^Cu and antibody assay by UV absorbance (280 nm).

Five radiolabelings of ^64^Cu-TETA-CD45 were performed. ^64^Cu in dilute HCl (5.8 to 7.5 mCi) was buffered with ammonium acetate, then added to CD45-TETA (0.2 to 0.8 mg) in 0.1 M ammonium acetate, pH 5 (0.12 to 0.27 mL). The radiolabeling solutions were incubated for 30 to 60 min at 37°C. Certified 0.1 M disodium ethylene diamine tetraacetic acid (EDTA) (Fisher Scientific) was added to a final concentration of 10 mM, to scavenge unchelated ^64^Cu. ^64^Cu-CD45-TETA was purified and transferred to PBS by molecular sieving filtration, and filtered. Radiochemical yield and product purity were determined by analytical HPLC of EDTA-challenged radiolabeling solution and purified ^64^Cu-TETA-CD45, respectively. The mean radiochemical yield was 75%, and all doses exceeded 95% radiochemical purity, with a mean activity of 17 mCi per mg of antibody.

Mouse anti-human CD45 antibody was conjugated to the zirconium-chelating agent desferrioxamine by combining antibody and desferrioxamine-p-SCN (Df-p-SCN) (Macrocyclics Inc., Dallas, TX) at concentrations of 32–45 µM and 200–430 µM, respectively, in 0.1 M sodium carbonate, pH 9 (0.35 to 0.51 mL) [9]. The molar ratios of Df-p-SCN to antibody were 6 to 12. The conjugation solutions were incubated for 1–2 h at 40°C. Df-CD45 was purified and transferred to 0.1 M HEPES, pH 7, by molecular sieving filtration. One to two Df molecules were conjugated per antibody, as determined by zirconium-binding assay with carrier-added ^89^Zr and antibody assay by UV absorbance (280 nm).

Five radiolabelings of ^89^Zr-Df-CD45 were performed. ^89^Zr in 1 M oxalic acid (2.2 to 4.4 mCi) was buffered with 2 M sodium carbonate, then added to Df-CD45 (0.40 to 0.66 mg) in 0.2 M HEPES, pH 7 (0.39 to 0.69 mL), The radiolabeling solutions were incubated for 1–2 h at room temperature. EDTA was added to a final concentration of 5 mM, to scavenge unchelated ^89^Zr. ^89^Zr-Df-CD45 was purified and transferred to saline by molecular sieving filtration, and filtered. Radiochemical yield and product purity were determined by analytical HPLC of EDTA-challenged radiolabeling solution and purified ^89^Zr-Df-CD45, respectively. The mean radiochemical yield was 89%, and all doses exceeded 95% radiochemical purity, with a mean activity of 4.2 mCi per mg of antibody.

### hPBSC Radiolabeling

Cell binding, internalization, and potential toxicity of ^64^Cu and ^89^Zr immunoconjugates were assessed in triplicate as described below. Briefly, ∼6×10^6^ hPBSC/mL were incubated with the immunoconjugate for 1 h (37°C) and cells washed three times with PBS, then radioactivity in the wash buffer and cell pellet counted. To assure that ^64^Cu or ^89^Zr was not released over time, the radiolabeled hPBSC were incubated in rhesus monkey serum under physiologic conditions, and the fraction of radioactivity associated with the cells measured at time points out to 3 (^64^Cu) and 7 (^89^Zr) days. The following assessments were performed in triplicate: (1) incubation time (hourly for 1–4 h); (2) amount of radioactivity for the incubation (µCi/cells/mL); (3) viability of the cells after incubation and over 48 h (trypan blue); and (4) efflux of radioactivity. Post-radiolabeling hPBSC were assessed in triplicate in hematopoietic colony forming unit (CFU) assays in parallel with unlabeled cells using established protocols [5].

hPBSC were incubated at room temperature for 30 min with ^64^Cu-TETA-CD45 or ^89^Zr-Df-CD45 at concentrations of 0, 20, 40, 80, or 160 µCi/mL in triplicate. A gamma count was taken on radiolabeled cells and medium obtained from the final wash with cell viability assessed using trypan blue. After radiolabeling, cells were incubated for 1 week at 37°C and 5% CO_2_. Cell counts were obtained for each dose with proliferation assessed by determining population doubling times using established protocols [5]. Radiolabeled cells were also plated in Methocult and incubated for 10 days for CFU assay. Differentiation potential of radiolabeled cells was assessed by counting erythroid (burst forming unit-erythroid, BFU-E) and myeloid (CFU-granulocyte macrophage, CFU-GM) colonies [5], [7]. A theoretical upper limit of radiolabeling efficiency was determined using QuantiBRITE™ PE beads following the manufacturer's protocol.

### *In vivo* Imaging

BLI was performed according to established protocols [10], [11] once animals were sedated with telazol and after an intravenous injection of 100 mg/kg D-Luciferin using a Xenogen IVIS®200 Imaging System with Living Image Software analysis (Caliper Life Sciences, Alameda, CA). Each animal was imaged using four views (anterior-posterior, posterior-anterior, right and left lateral) at each imaging session in a light tight chamber, and whole body images obtained with quantification performed. Bioluminescence and photographic images were superimposed using Living Image 2.50 software. Regions of interest (ROIs) were defined by selecting areas showing bioluminescence. Numbers of total photons/sec/cm^2^ detected in ROIs were recorded.

The microPET P4 imaging system (Siemens Preclinical Solutions, Inc., Malvern, PA) has a 22 cm bore, 20 cm transaxial field of view, and 8 cm axial field of view, and sensitivity of the scanner is 2.25% at the center of the field of view with an energy window of 250–750 keV and a timing window of 10 ns (default values) [12]. With maximum a posteriori (MAP) reconstruction incorporating an accurate system model (standard reconstruction algorithm used), image resolution is ∼1.8 mm isotropically (6 µL volumetric resolution). PET signals were analyzed and quantified with AMIDE (amide.sourceforge.net) software.

Animals were sedated with telazol and supplemented with ketamine as needed and injected intravenously with radiolabeled hPBSC (N = 2) or ∼0.3 mCi of ^64^Cu-TETA-CD45 or ^89^Zr-Df-CD45 (N = 4; 2 per radioimmunoconjugate). Each animal was placed on the scanner bed (supine) and the upper abdominal area was positioned in the center field of view based on images obtained by BLI. Static PET scans were acquired for ∼60 min on day 0, day 2, day 5, and day 9 post-injection. All listmode data were sorted into 3D sinograms using a span of 3 and a ring difference of 31. Images were reconstructed with a 2D OSEM reconstruction algorithm with an imaging matrix of 128×128×112 with a corresponding voxel size of 0.19×0.19×1.21 mm^3^.

### Statistical Analysis

Results are reported as the mean ± standard error of the mean and calculated using Microsoft Excel (Microsoft, Redmond, WA). Statistical significance (*p*<0.05) was determined by analysis of variance or two-sided Student's *t*-test analysis.

## Results

### ^64^Cu-CD45 and ^89^Zr-CD45 Immunoconjugates

To investigate the feasibility of tracking cells after transplantation with PET, optimal conditions were explored using a human-specific CD45 antibody and radiolabeling hPBSC with either ^64^Cu-TETA-CD45 or ^89^Zr-Df-CD45. In a preliminary study the selected CD45 clone HI30 showed a very high specificity for human cells with no binding to rhesus monkey cells. The CD45 antibody was successfully conjugated to ^64^Cu or ^89^Zr after several *in vitro* studies.

hPBSCs radiolabeled with each of the immunoconjugates were incubated with secondary antibodies against CD45 for analysis by flow cytometry. Results showed no significant differences in the percentage and mean fluorescence of hPBSC labeled with the immunoconjugates when compared to control cells (data not shown). Thus, no internalization was observed.

### Optimum Labeling Dose

Cryopreserved hPBSC were thawed and plated in 12-well culture plates at 1×10^6^ cells per well in X-VIVO 15™ medium supplemented with SCF, TPO, and FLT3L (50 ng/mL each) as noted above. Cells were incubated at room temperature for 30 min with ^64^Cu-TETA-CD45 or ^89^Zr-Df-CD45 with the range of concentrations identified.

**Figure 1:**
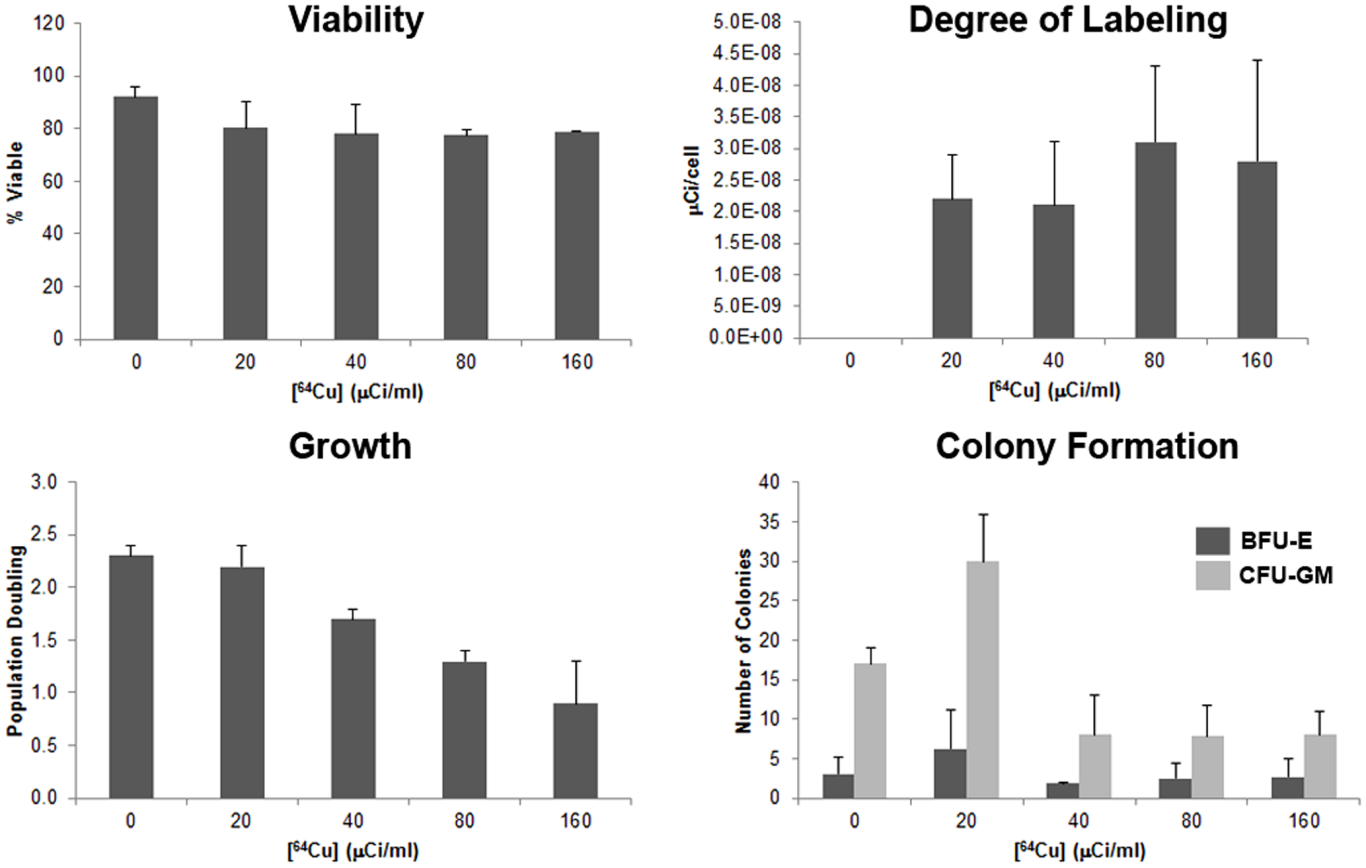
^64^Cu-TETA-CD45 radiolabeling of hPBSC. hPBSC were radiolabeled with 0, 20, 40, 80, or 160 µCi/mL of ^64^Cu-TETA-CD45. No significant changes in cell viability or degree of labeling were observed with increasing concentrations. A decline in cell growth and colony formation was observed when cells were incubated with ^64^Cu-TETA-CD45 at a concentration >20 µCi/mL.

For ^64^Cu, no significant decrease in viability was observed as the dose of ^64^Cu-TETA-CD45 increased from 0 (92%) to 160 µCi/mL (76%; *p*>0.05) (Figure 1). Radiolabeling efficiency was not significantly increased with concentrations of ^64^Cu-TETA-CD45 greater than 20 µCi/mL (*p*>0.5). For 0, 20, 40, 80, and 160 µ/mL, activities were 0.003, 0.023, 0.022, 0.031, and 0.028 pCi/cell, respectively. Toxicity affecting both growth and colony formation (BFU-E, CFU-GM) was observed with cells radiolabeled with ≥40 µ/mL (*p*<0.05). Population doublings observed for 0, 20, 40, 80, and 160 µCi/mL were 2.3±0.1, 2.2±0.2, 1.7±0.1, 1.3±0.1, and 0.9±0.4, respectively. CFU-GM identified at each of the doses were 17.1±2.1, 30.2±6.1, 8.4±5.3, 7.8±4.2, and 8.0±3.1, respectively. No toxicity was observed in BFU-E colony formation. Peak theoretic radiolabeling efficiency was calculated to be 10-fold more (0.32 pCi/cell) than the greatest measured radioactivity per cell, suggesting further optimization may be able to be achieved.

**Figure 2:**
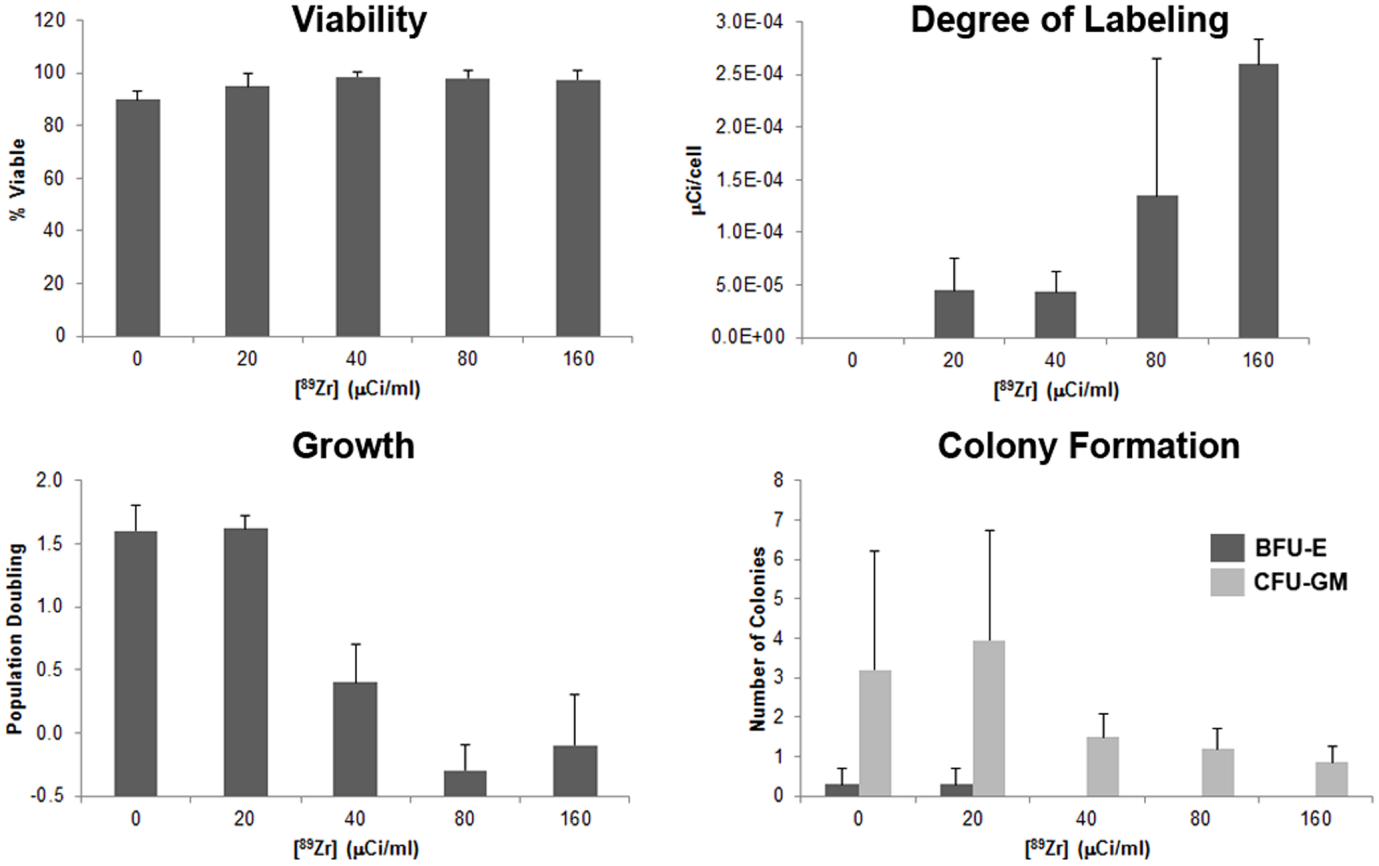
^89^Zr-Df-CD45 radiolabeling of hPBSC. hPBSC were labeled with 0, 20, 40, 80, or 160 µCi/mL of ^89^Zr-Df-CD45. No significant changes in cell viability were observed with increasing concentrations. However, a greater degree of radiolabeling was observed with 80 and 160 µCi/mL of ^89^Zr-Df-CD45. A decline in cell growth and colony formation was noted when cells were incubated with ^89^Zr-Df-CD45 at a concentration >20 µ/mL.

For ^89^Zr, there were no significant differences in viability observed between the dose groups evaluated (*p*>0.05) (Figure 2). As shown in Figure 2, a trend in increasing radiolabeling efficiency was observed as the dose of ^89^Zr-Df-CD45 increased with a significantly greater level of radiolabeling for 160 µCi/mL compared to 0–40 µ/mL (*p*<0.05). A greater than 2,000 fold-increase in the level of ^89^Zr-Df-CD45 labeling efficiency was observed when compared to ^64^Cu-TETA-CD45. Similar to the study using ^64^Cu-TETA-CD45, toxicity was observed with cells radiolabeled with ≥40 µCi/mL (*p*<0.05) when growth and colony formation (BFU-E, CFU-GM) were evaluated. Taken together, 20 µCi/mL showed the highest level of efficiency without altering cell function.

### Biodistribution of Immunoconjugates

**Figure 3:**
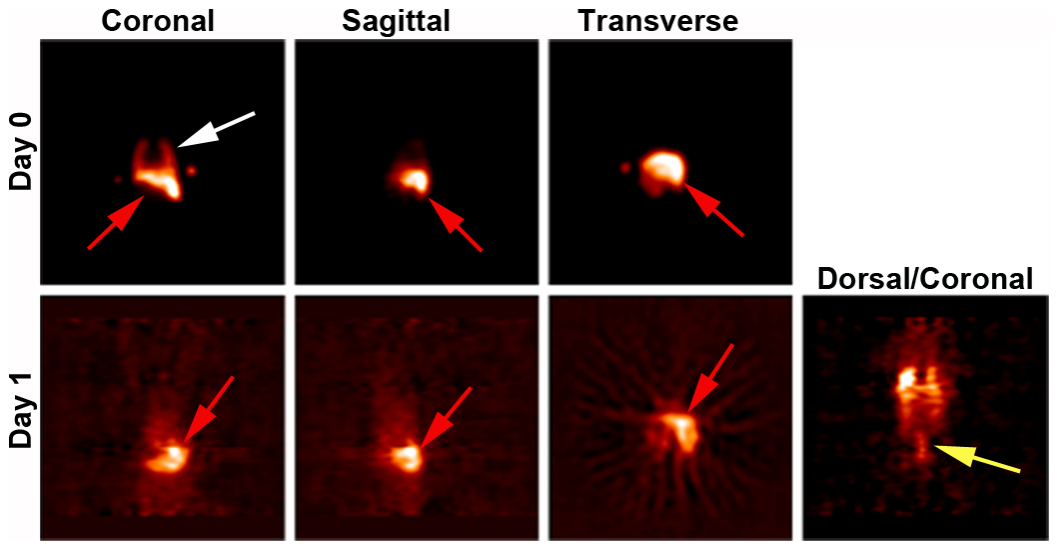
Short-term tracking of hPBSC radiolabeled with ^89^Cu-PTSM by PET. hPBSC were radiolabeled with 20 µCi/mL of ^64^Cu-PTSM. Cells radiolabeled with ^64^Cu-PTSM were detected in the lung (white arrow) and liver (red arrow) on the day of postnatal transplant (day 0). Cells were observed in the liver (red arrow) and spinal column (yellow arrow) 24 h post-injection (day 1).

Based on outcome, *in vivo* studies with ^64^Cu-TETA-CD45 were not pursued because the per-cell activity was significantly lower when compared to ^89^Zr-Df-CD45. When these results were compared to our prior studies with ^64^Cu-PTSM, a greater level of cell labeling was noted which may be related to PTSM which labels the cells by passive diffusion [5] in contrast to radioimmunoconjugates that target the cell surface. The half-life of ^64^Cu is also relatively short (12.7 days) thus allowing 3 days of imaging whereas ^89^Zr has a half-life of 3.3 days allowing cells to be tracked for approximately 2 weeks. Two animals injected with 5×10^6^ cells radiolabeled with ^64^Cu-PTSM at 20 µCi/mL showed signals in the lung (white arrow) and liver (red arrow) immediately post-injection (Figure 3). Radioactivity was also detected in the lumbar spine (yellow arrow). Animals showed no detectable level of radioactivity in subsequent scanning sessions. For short-term cell trafficking studies, 5×10^6^ hPBSC radiolabeled with ^89^Zr-Df-CD45 at the optimal dose of 20 µCi/mL were injected intravenously (N = 2), and animals were imaged 24 and 48 h post-injection, then 1 week after transplantation. No detectable PET signals were observed immediately following injection (data not shown). These studies suggest that ^64^Cu-PTSM may be more efficient for direct radiolabeling hPBSC and *in vivo* detection.

### Efficiency of ^89^Zr-CD45 Immunoconjugate to Identify Engrafted Human Cells

**Figure 4:**
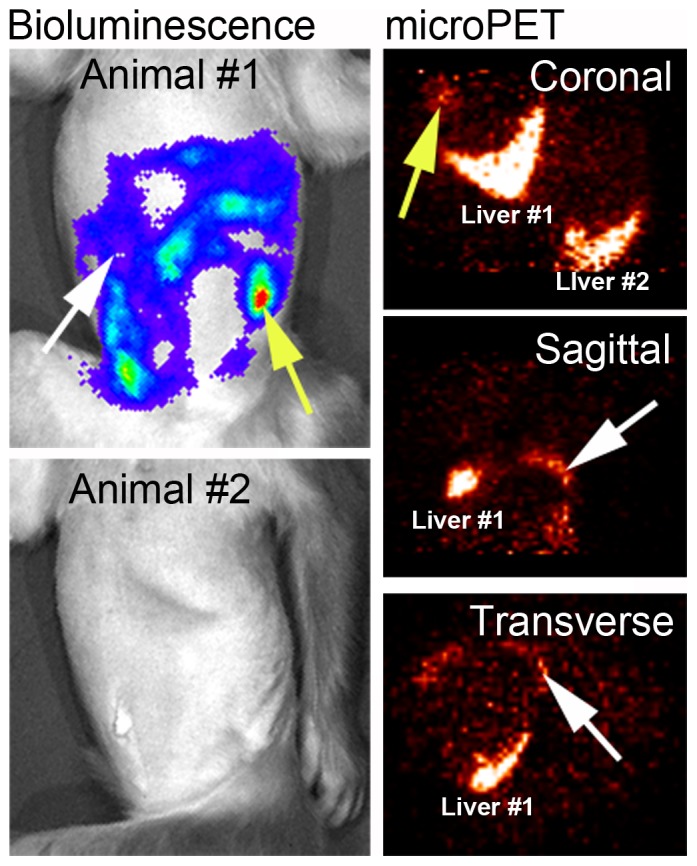
BLI and PET imaging of transplanted hPBSC. hPBSC expressing firefly luciferase were transplanted prenatally in the late first trimester [7]. At ∼3 months postnatal age D-luciferin was injected intravenously and BLI was performed to confirm the anatomical location of transplanted cells, then ^89^Zr-Df-CD45 was injected. Monkey #1 showed a high level of bioluminescence in the abdominal region. Both animals showed strong PET signals on day 5 post-injection of ^89^Zr-Df-CD45 within the liver. The muscular component of the peritoneum (white and yellow arrows) of Monkey #1 showed corresponding BLI and PET signals.

The CD45 antibody was directly bound to ^89^Zr and used as a method to identify engrafted cells that had previously been confirmed by BLI (prenatal hPBSC transplant, postnatal imaging). Animals transplanted prenatally with 25×10^6^ hPBSC expressing firefly luciferase were injected with 0.3 mCi of ^89^Zr-Df-CD45 intravenously at 3 months postnatal age and PET imaging performed at 0, 1, 3, 5, and 9 days post-injection (N = 2). Monkey #1 showed a high level of bioluminescence (3.0×10^7^ p/s) in the right abdomen whereas very low signals (2.1×10^6^ p/s) were observed for Monkey #2 (Figure 4). As anticipated, high background PET signals were observed in the liver prior to day 5 (data not shown). PET imaging on day 5 post-injection showed CD45 labeling (yellow arrow) within the peritoneum of Monkey #1, which was consistent with the corresponding BLI image. Monkey #2 showed signals in the liver with no detectable radioactivity observed on day 9 post-injection.

## Discussion and Conclusions

These studies focused on new methods for the development of CD45 immunoconjugates with ^64^Cu and ^89^Zr. Nontoxic hPBSC radiolabeling conditions were identified and an optimal dose selected for *in vivo* imaging studies. The outcome of these preliminary investigations suggest that the ^64^Cu immunoconjugate was not as efficient when compared to radiolabeling cells with ^64^Cu-PTSM, and that the signal from intravenously injected radiolabeled hPBSC (using either immunoconjugate) was below the level of detection by the microPET scanner used in these studies. Outcomes also suggest that the direct injection of ^89^Zr-Df-CD45 was the most efficient in identifying engrafted hPBSC in the rhesus monkey host. While these studies were conducted in a small group of animals, they report for the first time new stem/progenitor cell radiolabeling methods *in vitro* and the potential applications *in vivo*.

We have previously reported the optimization of radiolabeling rhesus monkey stem and progenitor cells with ^64^Cu-PTSM without evidence of radiotoxicity [5]. PTSM is a lipophilic redox-active carrier molecule that can deliver ^64^Cu into the cell passively. When Cu(II)-PTSM is reduced to Cu(I)-PTSM, Cu(I) is captured by intracellular macromolecules while the neutral PTSM diffuses out of the cell. This method has been shown to result in efficient intracellular radiolabeling of target cells [5]. However, radiotoxic Auger electrons have a penetration of 0.02–10 µm with very high DNA toxicity within this range. ^64^Cu emits a 6.84-keV Auger electron with a penetration range of approximately 5 µm and can potentially result in DNA damage, thus, requiring a lower dose for labeling to prevent radiotoxicity. Thus, in this study, a human-specific antibody to a membrane protein, a pan-hematopoietic marker CD45, was used for radiolabeling rather than the intracellular approach with PTSM. We hypothesized that labeling of the cell membrane using the human-specific CD45 antibody conjugated to ^64^Cu or ^89^Zr would increase radiolabeling efficiency while minimizing radiotoxicity. However, no increase in radiolabeling efficiency was observed in these studies. In addition, the antibody-based membrane labeling approach showed a similar level of radiotoxicity compared to the intracellular PTSM radiolabeling method. These findings suggest that the concentration of radioisotope in the labeling solution is an important factor in determining radiotoxicity when compared to the quantity and location of ^64^Cu bound to cellular components. Similar findings were observed when cells were radiolabeled using the CD45 antibody conjugated to ^89^Zr.

In our *in vitro* radiolabeling studies, CD45 antibodies conjugated to ^89^Zr consistently resulted in a higher labeling efficiency compared to ^64^Cu at the same concentration of radiotracers. When hPBSC radiolabeled with the CD45 antibody and ^89^Zr were directly injected *in vivo* and subsequently imaged with PET, no detectable signals were observed above background. For comparison, hPBSC radiolabeled with ^64^Cu-PTSM showed clear signals in the lung and spinal column, with high background in the liver. These findings suggest that there are insufficient CD45 molecules on the plasma membrane, which were estimated to be 1.38×10^5^ antibody binding sites per cell (unpublished findings), to provide sufficient antibody binding for radiolabeling and PET imaging. However, this finding does not preclude the possibility of identifying another membrane-bound molecule that is present at a higher frequency than CD45 for radiolabeling purposes. In addition, a polyclonal antibody with a greater number of binding sites per target molecule could potentially be explored, and a combination of these factors may enhance radiolabeling efficiency.

Current instrumentation and methodology for PET is optimized for detecting and quantifying relatively high levels of radiotracer concentrations that are distributed throughout the body. The most common example is ^18^F-fluorodeoxyglucose (FDG) for imaging glucose metabolism. The imaging environment for cell studies *in vivo* is vastly different. The total amount of radioactivity inside the subject is typically 3 orders of magnitude lower (µCi versus mCi), and the distribution of this activity is sparse and confined to cell location. Therefore, issues related to background become critical in identifying very weak signals, and the image reconstruction methodologies typically employed have not been optimized for this task. A major limitation of commercially available PET scanners is the use of detector materials that incorporate lutetium that has a naturally occurring isotope, Lu-176, which makes the detectors slightly radioactive. We have focused on this potential limitation by comparing PET scanners based on the scintillator lutetium oxyorthosilicate (LSO) and bismuth germanate (BGO) [13]. These related investigations have determined that radioactive LSO-based PET scanners do not perform as well when compared to those with non-radioactive materials such as BGO for imaging low activity sources. Optimizing parameters such as energy windows on the LSO-based scanners was also found to only lead to marginal improvements.

In conclusion, these studies report methods for developing CD45 immunoconjugates with ^64^Cu and ^89^Zr. Preliminary imaging studies also indicate that the ^64^Cu immunoconjugate was not as efficient for *in vivo* imaging when compared to radiolabeling cells with ^64^Cu-PTSM and the signal from injected radiolabeled hPBSC (using either immunoconjugate) was below the level of detection by the microPET scanner used in these studies. Of the approaches tested, direct injection of ^89^Zr-Df-CD45 was found to be the most efficient in identifying engrafted hPBSC. Additional studies with immunoconjugates and related cell labeling techniques are important in order to develop sensitive and safe methods to monitor cell trafficking and cell fate for future human application.

## References

[1] Pecking AP, Bellet D, Alberini JL (2012) Immuno-SPET/CT and immuno-PET/CT: a step ahead to translational imaging. Clin Exp Metastasis 29: 847–852. 22760521 10.1007/s10585-012-9501-5

[2] Rizvi SN, Visser OJ, Vosjan MJ, van Lingen A, Hoekstra OS, et al (2012) Biodistribution, radiation dosimetry and scouting of ^90^Y-ibritumomab tiuxetan therapy in patients with relapsed B-cell non-Hodgkin's lymphoma using ^89^Zr-ibritumomab tiuxetan and PET. Eur J Nucl Med Mol Imaging 39: 512–520. 22218876 10.1007/s00259-011-2008-5 PMC3276758

[3] Wallhaus TR, Lacy J, Stewart R, Bianco J, Green MA, et al (2001) Copper-62-pyruvaldehyde bis(N-methyl-thiosemicarbazone) PET imaging in the detection of coronary artery disease in humans. J Nucl Cardiol 8: 67–74. 11182711 10.1067/mnc.2001.109929

[4] Zhang Y, Hong H, Cai W (2011) PET tracers based on Zirconium-89. Curr Radiopharm 4: 131–139. 22191652 10.2174/1874471011104020131 PMC3246366

[5] Huang J, Lee CCI, Sutcliffe JL, Cherry SR, Tarantal AF (2008) Radiolabeling rhesus monkey CD34+ hematopoietic and mesenchymal stem cells with ^64^Cu-pyruvaldehyde-bis(N4-methylthiosemicarbazone) for microPET imaging. Mol Imaging 7: 1–11. 18384718

[6] Tarantal AF, Lee CCI, Batchelder CA, Christensen JE, Cherry SR (2012) Radiolabeling and *in vivo* imaging of transplanted renal lineages differentiated from human embryonic stem cells in fetal rhesus monkeys (*Macaca mulatta*). Mol Imaging Biol 14: 197–204. 21479709 10.1007/s11307-011-0487-1 PMC4224287

[7] Tarantal AF, Goldstein O, Barley F, Cowan MJ (2000) Transplantation of human peripheral blood stem cells (PBSC) into fetal rhesus monkeys (*Macaca mulatta*). Transplantation 69: 1818–1823. 10830217 10.1097/00007890-200005150-00015

[8] Tarantal AF. (2005) Ultrasound imaging in rhesus (*Macaca mulatta*) and long-tailed (*Macaca fascicularis*) macaques: Reproductive and research applications. In: The Laboratory Primate. ED. Wolfe-Coote S Amsterdam: Elsevier, 317–352.

[9] Meijs WE, Herscheid JDM, Haisma HJ, Pinendo HM (1992) Evaluation of desferal as a bifunctional chelating agent for labeling antibodies with Zr-89. Appl Radiat Isot 43: 1443–1447. 10.1016/0883-2889(92)90170-j 1334954

[10] Tarantal AF, Lee CC, Jimenez DF, Kohn DB, Cherry SR (2006) Fetal gene transfer using lentiviral vectors: *In vivo* detection of gene expression by microPET and optical imaging in fetal and infant monkeys. Hum Gene Ther 17: 1254–1261. 17134373 10.1089/hum.2006.17.1254

[11] Tarantal AF, Lee CCI (2010) Long-term luciferase expression monitored by bioluminescence imaging after adeno-associated virus-mediated fetal gene delivery in rhesus monkeys (*Macaca mulatta*). Hum Gene Ther 21: 143–148. 19751148 10.1089/hum.2009.126 PMC2829449

[12] Tai YC, Chatziioannou AF, Siegel S, Silverman RW, Meadors K, et al (2001) Performance evaluation of the microPET P4: A PET system dedicated to small animal imaging. Phys Med Biol 46: 1845–1862. 11474929 10.1088/0031-9155/46/7/308

[13] Freedenberg M, Badawi RD, Tarantal AF, Cherry SR. (2013) Performance and limitations of positron emission tomography (PET) scanners for imaging very low activity sources. Phys Med doi:pii: S1120-1797(13)00051-3. 10.1016/j.ejmp.2013.04.001 PMC3795820 23680361

